# Communicable Animal Diseases1Read before the twenty-second annual meeting of the New Jersey State Sanitary Association at Lakewood, N. J., December 12, 1896.

**Published:** 1897-02

**Authors:** W. Herbert Lowe

**Affiliations:** Paterson, N. J., Formerly United States Veterinary Inspector of the Port of New York


### COMMUNICABLE ANIMAL DISEASES.[1]

By W. Herbert Lowe, D.V.S.,
PATERSON, N. J.,
FORMERLY UNITED STATES VETERINARY INSPECTOR OF THE PORT OF NEW YORK.

(Concluded from page 21.)

The caring for the contagious animal diseases in this State has during past years been intrusted to the State Board of Health, and it is hardly necessary for me to allude to the effectiveness of the untiring efforts of our deceased fellow-laborer, the lamented Dr. Ezra M. Hunt. The good work inaugurated by Dr. Hunt is still being carried on and extended in many ways by the present able Secretary of the State Board of Health and his co-laborers. This board was successful last year in suppressing anthrax in Cumberland County and elsewhere in this State, and I am pleased to say that there has been no return of the disease during the present year. Forty-five cases of glanders were reported, of which number five cases, upon examination, showed no evidences of the disease. All the other cases were destroyed and action taken to prevent extension of the disease. The mallein-test has been employed as a diagnostic agent in suspicious cases with satisfactory results. In speaking of glanders I am reminded of a farcy case presenting some dangerous and alarming features that came under my observation some two or three months ago. A poor man bought a horse with sores on its legs, being told by the seller that the sores were only fly-bites. His twelve-year-old daughter bathed the sores for a week or more, when I was called. I explained to the parents the nature of the disease in the horse and the danger of inoculation to the girl, and advised treatment. Fortunately the girl has shown no symptoms of the disease, and as two or three months have elapsed it is probable that she was not inoculated with the virus. The limits of my paper are such that I must pass on rapidly. But before doing so let me state that the investigation and control of one single infectious disease have assumed such importance in our State from both a sanitary and from a financial standpoint that the Legislature two years ago, if I remember rightly, took the control of it out of the hands of the State Board of Health and passed a law creating a special commission on tuberculosis in animals. This commission unfortunately is limited in power and funds. But nothing has

[1] Read before the twenty-second annual meeting of the New Jersey State Sanitary Association at Lakewood, N. J., December 12, 1896.

prevented them from prosecuting the duties imposed upon them to the full extent of the means at their command. Under the statute they can only act when requested by the State Dairy Commissioner, the State Board of Health, or at the request of the owner. They have no power under the law of their own volition to examine any herd, no matter what its condition may be, without the request or consent of one of the forementioned.

When the commission organized and commenced its work some individuals were strongly opposed to it. These people mostly did not understand the insidious, chronic nature of tuberculosis, and that it was transmissible to the human family. Others, from selfish motives, were afraid that it would hurt their dairy business, and so on.

The commission, however, soon proved itself equal to the exigencies of the work. Its experienced and able Secretary, the Hon. Franklin Dye, inaugurated what was essentially "a campaign of education". His far-seeing intelligence told him that the only real impediment to the ultimate work of the commission was ignorance on the part of dairymen, cattlemen, and others of the essential nature and danger, not only to the animals, but to the health and wealth of the people themselves. When we consider that tuberculosis kills more people than any other disease among mankind, and that the tubercle bacillus of tuberculosis is transmissible from the cow, the importance of the question that confronts us becomes more evident from a sanitary standpoint.

The commission is a most conservative and safe body, moving slowly but surely, doing effective work. When called upon it inspects an infected herd, making a careful physical examination of each individual animal, and testing with tuberculin any cases that cannot be diagnosticated without its aid. Diseased animals are condemned, appraised, killed, and autopsies made, and owners are paid three-fourths of the appraised value. Premises are disinfected. The matters of proper ventilation, sunlight, and good sanitary conditions in general are encouraged as far as possible.

No public work where so many interests are involved was ever conducted without criticism and opposition to some extent; but I must say that I am surprised at the support given the New Jersey State Tuberculosis Commission by producers and consumers. Both see the importance and value of healthful food-products, and I have yet to meet the man who has had dealings with the commission who was not well satisfied and was not ready to support the furtherance of the work.

The "campaign of education" has been already productive of much good. The preliminary work so far has been judicious, careful, and thorough. It does not speak well for the people that this commission should be hampered in the important trust that has been committed to its care. I therefore bespeak for the commission that the Legislature be asked to enact necessary amendatory legislation and appropriate sufficient funds to prosecute successfully the work in hand.

Before concluding these fragmentary remarks I would invite your attention to what I personally believe to be a very important phase of the subject from an economic standpoint, which I referred to quite fully in an address recently delivered before the County Veterinary Medical Association of New York, at the New York Academy of Medicine. I refer to the necessity of breeding animals of greater constitution, stamina, and vigor. The fact that tuberculosis is caused by the tubercle bacillus, and is therefore not hereditary, does not lessen the importance of breeding dairy cattle having substance and stamina sufficient to resist infection and development of the micro-organism.

We all know that in time of war a city that is not properly fortified is easily taken by the enemy. So the animal body, if not fortified with a strong constitution and stamina, will be readily invaded by the micro-organisms of infections. The proper pabulum or soil for the development of microbes is only second in importance to the microbe itself. All persons exposed to infection of tuberculosis, smallpox, or scarlet fever do not take the disease because all are not alike susceptible. All men who eat the flesh or drink the milk of tuberculous cows do not contract tuberculosis. Whatever in any degree weakens or impairs the system of men or animals renders them liable to the invasion of disease. The animal that inherits a strong, untainted constitution and good health from its progenitors possesses a disease-resisting power that often means an immunity. If this principle of heredity was kept in mind in breeding dairy cattle, there would not be so much tuberculosis. Environment, sanitary conditions, climate, and food have also important influences upon the causation and development of tuberculosis, which department of the subject I hope to be able to treat more fully upon some future occasion. There is little benefit to be derived from destroying affected animals if you keep on breeding weak, delicate, and deteriorated cattle, which, when developed and forced to their full milking capacity, and stabled in a crowded, ill-ventilated, damp, dark, filthy, and unsanitary condition, will not be able to resist the dreaded micro-organism.

This breeding problem promises great and far-reaching results, if intelligently carried out by our farmers and breeders. In the human family no such theory could be carried out, but in the animal kingdom there is nothing to prevent. I trust that the matter will receive the consideration and attention that it deserves.

